# When fats commit crimes: fatty acid metabolism, cancer stemness and therapeutic resistance

**DOI:** 10.1186/s40880-018-0317-9

**Published:** 2018-07-11

**Authors:** Ching-Ying Kuo, David K. Ann

**Affiliations:** 1 Department of Clinical Laboratory Sciences and Medical Biotechnology, College of Medicine, National Taiwan University, Taipei, 10048 Taiwan China; 2 Department of Diabetes Complications and Metabolism, Diabetes and Metabolism Research Institute, Beckman Research Institute, City of Hope, Duarte, CA 91010 USA; 3 Irell and Manella Graduate School of Biological Sciences, City of Hope, Duarte, CA 91010 USA

**Keywords:** Fatty acid synthesis, Fatty acid oxidation, Fatty acid metabolism, Lipogenic phenotype, Cancer stem cells, Tumor-initiating cells, Cancer cell plasticity, Therapeutic resistance, Drug-tolerant persisters

## Abstract

The role of fatty acid metabolism, including both anabolic and catabolic reactions in cancer has gained increasing attention in recent years. Many studies have shown that aberrant expression of the genes involved in fatty acid synthesis or fatty acid oxidation correlate with malignant phenotypes including metastasis, therapeutic resistance and relapse. Such phenotypes are also strongly associated with the presence of a small percentage of unique cells among the total tumor cell population. This distinct group of cells may have the ability to self-renew and propagate or may be able to develop resistance to cancer therapies independent of genetic alterations. Therefore, these cells are referred to as cancer stem cells/tumor-initiating cells/drug-tolerant persisters, which are often refractory to cancer treatment and difficult to target. Moreover, interconversion between cancer cells and cancer stem cells/tumor-initiating cells/drug-tolerant persisters may occur and makes treatment even more challenging. This review highlights recent findings on the relationship between fatty acid metabolism, cancer stemness and therapeutic resistance and prompts discussion about the potential mechanisms by which fatty acid metabolism regulates the fate of cancer cells and therapeutic resistance.

## Background

Fatty acid (FA) metabolism is composed of anabolic and catabolic processes that maintain energy homeostasis. FA synthesis, which converts various types of nutrients into metabolic intermediates, is essential for cellular processes such as maintaining cell membrane structure and function, storing energy and mediating signaling. Cells generate energy by breaking down FAs via FA oxidation (FAO), also known as β-oxidation [1, 2]. A loss of balance between FA synthesis and oxidation may result in inadequate FA levels, leading to lipid accumulation. Lipid accumulation has been observed in many types of cancer, including brain, breast, ovarian and colorectal cancers [3–5] and has recently drawn increased attention. This has motivated scientists to understand the molecular mechanisms by which FA metabolism participates in the pathophysiological processes of cancer.

Cancer stem cells (CSCs), also referred to as tumor-initiating cells (TICs), have been identified in many types of solid tumors and often result in tumor recurrence because of their self-renewal and tumorigenic properties. CSCs/TICs can be defined by in vitro tumorsphere formation assays and in vivo limiting dilution assays in conjunction with surface marker analyses [6, 7]. How CSCs originate remains under debate. Possible explanations are that: (1) adult stem cells acquire mutations to become malignant or (2) neoplastic, differentiated cells receive external stimuli and undergo reprogramming to a progenitor or stem-like state [8]. Recent findings on the interconversion of neoplastic epithelial cells to CSC-like cells within a mixed tumor population suggest that a dynamic reprogramming process may occur during the transition state [9–12]. This bidirectional conversion or so-called cancer cell plasticity may emerge as a challenge for cancer treatment [13, 14]. Therefore, delineating the mechanisms of cancer cell plasticity and identifying regulators of the process that can be manipulated to prevent the conversion of cancer cells to CSCs may reduce the incidence of cancer recurrence.

A similar idea applies to the development of therapeutic resistance. Cancer treatments typically kill most fast-growing tumor cells. However, a subpopulation of cells may become tolerant of the drug, enter a state of dormancy and later evolve mechanisms of resistance. The cells in this small population are called drug-tolerant persisters (DTPs) and are considered independent from cells that acquire mutations to develop resistance. The interconversion between the drug-sensitive state and the tolerant state is thought to be controlled by growth factor signaling or epigenetic regulation [15–18]. For example, DTPs arising from tyrosine kinase inhibitor-resistant lung cancer cells are regulated by insulin growth factor signaling and a lysine demethylase, KDM5A [18]. In addition, these DTPs express the stem cell marker CD133 and share some of the CSC properties. Therefore, when appropriate, CSCs/TICs/DTPs will be used hereafter to describe the small populations of cells possessing the abilities to confer drug resistance and to repopulate. Understanding the mechanisms by which cancer cells progress into CSCs/TICs/DTPs will offer opportunities to prevent therapeutic resistance.

The plasticity of cancer cells and the genetic-independent acquisition of therapeutic resistance may be tightly associated with metabolic reprogramming. Altered metabolism is one of the hallmarks of cancer and has also been observed in CSCs (reviews in [19–22]). Hirsch et al. have shown that metformin, a blood sugar-lowering drug specifically targets breast CSCs and sensitizes CSCs to doxorubicin [23]. Metformin not only activates AMP-activated kinase (AMPK), but also inhibits complex I of the mitochondrial respiratory chain [24], suggesting that CSCs may have distinct metabolic features that are targetable. A study reveals that the loss of fructose-1,6-biphosphatase (FBP1) in basal-like breast cancer inhibits oxidative phosphorylation (OXPHOS), increases glycolysis and CSC properties [25]. Moreover, mesenchymal glioma stem cells derived from clinical specimens demonstrate elevated glycolytic activity. In contrast, mitochondrial biogenesis and OXPHOS are also critical for maintaining CSC populations [26, 27]. These findings suggest that there is metabolic plasticity in the CSC population and that modulating the utilization of metabolic pathways could influence the tumorigenic capacity of tumor cells.

While increasing evidence has revealed the role of altered energy metabolism during cancer progression, relatively fewer studies have focused on FA metabolism. In this review, we aim to evaluate recent studies and to summarize their findings on the role of FA metabolism in cancer malignant phenotypes, especially therapeutic resistance and stemness. We wish to stimulate discussion of the mechanisms by which cancer cells may acquire malignant properties via altered FA metabolism.

## Fatty acid metabolism in cancer progression and therapeutic resistance

**Fig. 1:**
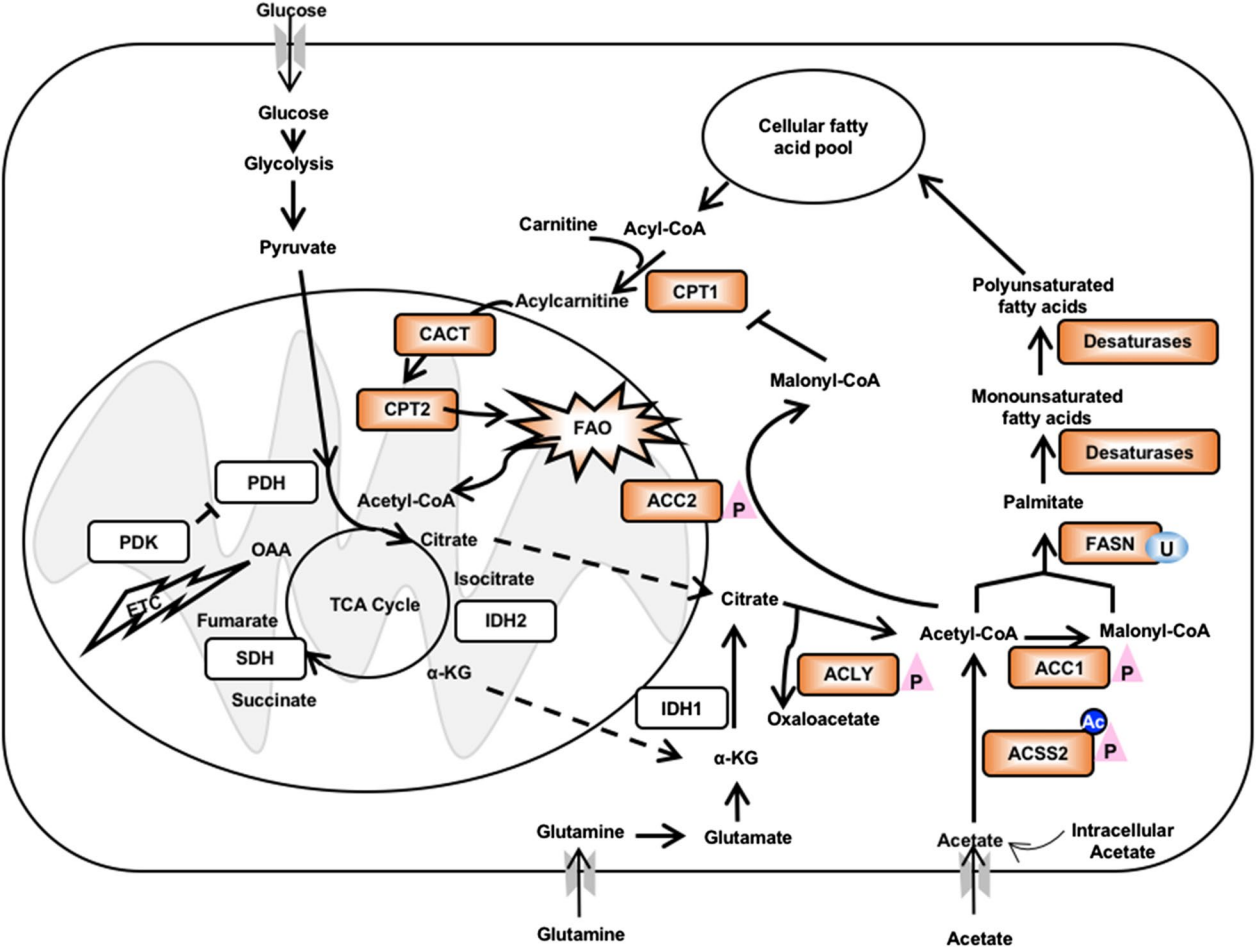
Fatty acid metabolism in cancer. Key enzymes involved in fatty acid (FA) metabolism. Orange-highlighted enzymes have been reported as altered in cancer or associated with cancer stemness. *ACC* acetyl-CoA carboxylase, *ACLY* ATP citrate lyase, *ACSS2* acyl-CoA synthetase short-chain family member 2, *FASN* fatty acid synthase, *CPT1*/*2* carnitine/palmitoyl-transferase 1/2, *CACT* carnitine acylcarnitine translocase, *FAO* fatty acid oxidation, *IDH* isocitrate dehydrogenase, *TCA cycle* tricarboxylic acid cycle, *PDK* pyruvate dehydrogenase kinase, *PDH* pyruvate dehydrogenase, *P* phosphorylation, *U* ubiquitylation, *Ac* acetylation

The lipogenic phenotype is one of the metabolic hallmarks of cancer. First observed in the 1950s, de novo FA synthesis is the major source of FAs for cancer cells [28]. Rapidly growing cancer cells require relatively large amounts of FAs to support processes such as membrane formation and signaling. Cytosolic acetyl-CoA is the building block for FAs, and can be generated from citrate or acetate. Citrate comes from either glycolysis followed by the tricarboxylic acid (TCA) cycle or from glutaminolysis followed by reductive carboxylation; it is then cleaved by ATP-citrate lyase (ACLY) to form cytosolic acetyl-CoA and oxaloacetate. Acetate obtained from either external or internal sources is ligated to CoA by acyl-CoA synthetase short-chain family member 2 (ACSS2) to form acetyl-CoA. Next, acetyl-CoA is carboxylated by acetyl-CoA carboxylase (ACC) to form malonyl-CoA. This is followed by a series of condensation processes catalyzed by fatty acid synthase (FASN) in the presence of nicotinamide adenine dinucleotide phosphate (NADPH) to primarily produce palmitate for subsequent FA elongation, desaturation and lipid synthesis [1, 29] (Fig. 1).

In tumors, many lipogenic enzymes are up-regulated and correlate with cancer progression (Fig. 1). Overexpression of *FASN* has been frequently reported in a wide variety of cancers, including breast, ovarian, endometrial and prostate cancers, and is associated with poor prognosis and resistance to chemotherapy [29–35]. For example, increased expression of *FASN* is associated with resistance to cisplatin in breast and ovarian cancers and the resistance can be reversed by blocking FASN with an inhibitor, C75 [30, 31]. FASN increases DNA repair activity by up-regulating poly(ADP-ribose) polymerase 1 resulting in resistance to genotoxic agents [35]. In cancer cells, expression of FASN is modulated by sterol regulatory element-binding protein 1c (SREBP1) and proto-oncogene *FBI*-*I* (Pokemon) via dysregulated mitogen activated protein kinase or phosphoinositide 3-kinase/AKT pathways under hormonal or nutritional regulation [1, 36]. FASN expression can also be regulated post-translationally. The deubiquitinase USP2a is often up-regulated and stabilizes FASN in prostate cancer [37].

ACLY serves as a central hub for connecting glucose and glutamine metabolism with lipogenesis and initiating the first step of FA synthesis [38]. Elevated *ACLY* levels have been observed in gastric, breast, colorectal and ovarian cancers and are linked to malignant phenotypes and poorer prognosis [39–42]. In particular, overexpression of *ACLY* in colorectal cancer leads to resistance to SN38, an active metabolite of irinotecan [42]. Like *FASN*, the transcription of *ACLY* is also regulated by SREBP1 [43], and it can be regulated post-translationally. Phosphorylation at ACLY serine 454 by AKT is increased in lung cancer and is correlated with enhanced activity of ACLY [44]. ACLY can also be phosphorylated by cAMP-dependent protein kinase and nucleoside diphosphate kinase [45, 46].

Overexpression of *ACC* has been found in breast, gastric and lung cancers [47–49]. Mammals express two isoforms of ACC, ACC1 and ACC2, which have distinct roles in regulating FA metabolism. ACC1 is present in the cytoplasm, where it converts acetyl-CoA to malonyl-CoA. ACC2 is localized to the mitochondrial membrane, where it prevents acyl-CoA from being imported into the mitochondria through carnitine/palmitoyl-transferase 1 (CPT1) for FAO and entering the TCA cycle to generate energy. Both ACC1 and ACC2 can be regulated transcriptionally and post-translationally by multiple physiological factors, including hormones and nutrients [50, 51]. mRNA expression of *ACC1* and *ACC2* is regulated by SREBP1, carbohydrate-responsive element-binding protein and liver X receptors [52, 53]. Additionally, ACC1 and ACC2 can be phosphorylated at serine 80 (serine 79 in mouse) and serine 222 (serine 212 in mouse), respectively, by tumor suppressor AMPK to inhibit their activities under ATP-depleted condition [50, 54–57]. The phosphorylation at serine 80 of ACC1 is associated with a metastatic phenotype in breast and lung cancers and is also responsible for resistance to cetuximab in head and neck cancer [58, 59].

There are 26 genes encoding acyl-CoA synthetase, which have distinct affinities for short-, medium-, long- or very long-chain FAs [60]. Overexpression of cytosolic ACSS2, one of the three family members of short chain acyl-CoA synthetase, can lead to acetate addiction in breast, ovarian, lung and brain cancers when nutrients or oxygen are limited; this overexpression is correlated with cancer progression and worse prognosis [61–63]. Mitochondrial ACSS1 is up-regulated in hepatocellular carcinoma and is associated with tumor growth and malignancy [64]. Although the regulation of *ACSS* expression remains poorly understood, it has been reported that *ACSS* genes are controlled by SREBP [65, 66].

In addition to the highly activated lipogenic pathway, FA catabolism is also important for maintaining cancer cell survival and contributing to chemotherapy resistance. The mitochondrial inner membrane is impermeable to long-chain acyl-CoAs; thus, the CPT system is required for transporting long-chain acyl-CoAs into the mitochondria from the cytoplasm. Three components are involved in this transporting system: CPT1, the carnitine acylcarnitine translocase (CACT) and CPT2 [67]. There are three currently known isoforms of CPT1 distributed in different tissues: CPT1A, CPT1B and CPT1C [68]. Knockdown of *CPT1A* leads to down-regulation of mTOR signaling and increases of apoptosis, suggesting CPT1A promotes the growth of prostate cancer cells [69]. Moreover, *CPT1A* depletion can sensitize prostate cancer cells to anti-androgen treatment, enzalutamide [70]. It has also been reported that CPT1A is positively correlated with histone deacetylase activity to enhance the tumorigenesis of breast cancer [71]. The expression of *CPT1A* can be regulated by nuclear receptors, PPARs and the PPARγ coactivator (PGC-1) [72]. PPARs have also been implicated as playing important roles in cancer progression [73]. CPT1A has also been shown to support the proliferation of leukemic cells and the knockdown or inhibition of CPT1A by a pharmacological inhibitor etomoxir (ETO) sensitizes leukemic cells to a chemotherapeutic drug, cytarabine [74]. In addition to *CPT1A*, AMPK regulates *CPT1C* expression to promote tumor growth upon metabolic stress in several types of cancer cells. Down-regulation of *CPT1C* enhances the sensitivity to mTOR inhibitor, rapamycin in cancer cells [75]. Only a few studies have reported the dysregulated *CPT1B* expression in colorectal and bladder cancers [76, 77]. A recent study has revealed the relationship between STAT3-induced *CPT1B* expression and chemoresistance in breast cancer cells [78].

In comparison to CPT1, relatively less studies have pointed out the roles of CPT2 and CACT in cancer. Knockdown of *CPT2* significantly impedes the growth of *MYC*-overexpressing triple-negative breast cancer (TNBC) cells [79]. Another report also shows that depletion of *CPT2* hinders TNBC growth via the down-regulation of the phosphorylated Src levels [80]. These data suggest an oncogenic role of CPT2 in TNBC. On the other hand, a meta-analysis has revealed that higher *CPT2* expression is correlated with better outcome in colorectal cancer patients [81]. CACT has been found to be overexpressed in prostate cancer cells and down-regulated in bladder cancer [76, 82]. Therefore, the exact role of CACT in cancer progression and therapeutic resistance remains uncertain.

## Fatty acid synthesis and cancer stemness

Similar to the expression patterns of lipogenic genes in cancer cells, several lipogenic genes are dysregulated in CSCs and are critical for CSC expansion and survival. However, how these genes are regulated in CSCs and why CSCs depend upon their lipogenic potential require further investigation. A recent study reported that glioma stem cells prefer to utilize glucose and acetate as carbon sources, compared with differentiated glioma cells [83]. In that study, FASN was concurrently expressed with glioma stem cell markers, including SOX2, CD133 and Nestin. In glioma stem cells, inhibition of FASN by the fatty acid synthesis inhibitor cerulenin decreases expression of glioma stem cell markers and reduces the number of tumorspheres formed [83]. In pancreatic CSCs, *FASN* is up-regulated and the inhibition efficacy of cerulenin is greater on pancreatic CSCs than on pancreatic cancer cells [84]. In breast CSCs, down-regulation of *FASN* by metformin via the induction of miR-193b leads to inhibition of mammosphere formation [85]. The antioxidant-like plant polyphenol resveratrol also decreases FASN to promote apoptosis in breast CSCs [86]. Taken together, these studies suggest that FASN is involved in promoting CSC survival.

*ACLY* also plays an important role in CSCs. In an in vitro lung cancer cell model, knockdown of *ACLY* inhibits epithelial–mesenchymal transition (EMT), a phenomenon often linked to cancer stemness, and results in a decrease of tumorsphere formation [87]. Treating MCF7 breast cancer cells with soraphen A, a specific inhibitor of ACC, significantly reduces the population of CSCs, as defined by CSC marker ALDEFLUOR. The effects of this inhibition are even greater in MCF7 cells overexpressing the proto-oncogene human epidermal growth factor receptor 2 (HER2) [88].

Elevated levels of unsaturated FAs have been observed in ovarian CSCs and it was recently reported that desaturases control the fate of ovarian CSCs [89, 90]. In these studies, inhibition of desaturases by CAY10566 or SC-26196 diminishes cancer stemness by reducing stemness markers, including SCD1, ALDH1A1 and SOX2. Blockade of FA desaturation impairs NF-κB signaling, which also directly regulates the unsaturation of FAs.

## FAO and cancer stemness

FAO is composed of a cyclical series of catabolic reactions and results in the shortening of fatty acids (two carbons per cycle). It is an essential source of reduced nicotinamide adenine dinucleotide (NADH), flavin adenine dinucleotide (FADH2), NADPH and ATP. NADH and FADH2 enter the electron transport chain to produce ATP, and NADPH protects cancer cells against metabolic stress and hypoxia [67]. As the key rate-limiting enzyme of FAO, CPT1 conjugates fatty acids with carnitine for translocation into the mitochondria; therefore, it controls FAO directly and thus facilitates cancer metabolic reprogramming. CPT1 also shares multiple connections with many other cellular signaling pathways often dysregulated in cancers, such as aerobic glycolysis, FAS, p53/AMPK axis, mutated RAS, mTOR and STAT3 [91, 92]. This evidence positions CPT1 as a multifunctional mediator in cancer pathogenesis and resistance to treatment.

In HER2-positive breast cancer cells, pharmacological inhibition of PPARγ by GW9662 results in a decrease in CSC number and down-regulated expression of CSC markers, presumably via increased production of reactive oxygen species (ROS) [93]. Whether the effects of PPARγ inhibition perturb the activity of FAO in HER2-positive breast CSCs remains unclear.

An interesting phenomenon has been observed in both leukemia and breast cancer. In leukemic cells, FAO is uncoupled from ATP synthesis and FA synthesis is enhanced to support FAO. Therefore, inhibiting FAO using ETO reduces the numbers of quiescent leukemic progenitors, which are able to initiate leukemia in the immune-deficient mice [74]. In breast cancer cells, prolonged treatment with the metabolic intermediate dimethyl α-ketoglutarate (DKG) leads to accumulation of succinate and fumarate, which induces hypoxia-inducible factor 1α (HIF-1α) to promote both glycolysis and OXPHOS to enable the plasticity of breast cancer cells. However, the increased OXPHOS is uncoupled from ATP synthesis and can be dampened by ETO, so the detected oxygen consumption presumably comes from FAO. Moreover, inhibiting both glycolysis and FAO by dichloroacetate and ETO respectively can decrease DKG-induced tumorsphere formation and accumulation of FAs was observed in DKG-treated breast cancer cells, suggesting that increased FAs may be utilized to support FAO [94]. It is likely that FA synthesis and FAO feed-forward with one another. Additional possible sources of FAs may come from reductive carboxylation [95, 96] or extracellular lysophospholipids through macropinocytosis [97]. Indeed, altered lipid metabolism appears to play a role in TNBC: TNBC and non-TNBC patient tissues can be discriminated based on markers of lipid metabolism [98, 99].

NANOG, a transcription factor and known stem cell marker, was recently reported to promote mitochondrial FAO in CSCs and support liver oncogenesis and drug resistance [100]. In that report, inhibition of FAO by ETO limits the expansion of CSCs and sensitizes CSCs to sorafenib kinase inhibitor treatment. In this case, it is possible that NANOG-positive cells become CSCs/TICs/DTPs to exert resistance to sorafenib. How NANOG regulates FAO and how FAO promote resistance warrant further investigation.

More recently, breast adipocyte-derived leptin was shown to activate JAK/STAT3 signaling through the leptin receptor to up-regulate *CPT1B*, leading to enhanced FAO in breast CSCs [78]. FAO is critical for maintaining breast CSCs and is associated with chemoresistance. Blocking FAO with perhexiline, an FDA-approved drug for treatment of angina and heart failure [101], can sensitize chemoresistant breast cancer cells to the mitotic inhibitor paclitaxel.

## Perspectives

CSCs/TICs, a minor population of cells capable of self-renewal and tumor initiation, are tightly associated with cancer relapse, metastasis and chemoresistance. The theory of CSC origin is currently based on two models: hierarchical and stochastic. The classic hierarchical model suggests that only a subset of cancer cells has the ability to self-renew and divide [8, 102]. On the other hand, accumulating evidence supports the stochastic model that every cancer cell has the potential to be reprogrammed into a CSC when the appropriate cues are present [9–12]. DTPs are a relatively new concept in cancer treatment resistance. This subpopulation is responsible for the development of drug resistance and shares similar properties with CSCs/TICs, but does not fully resemble them. The chromatin state is altered in DTPs [18], suggesting that the chromatin has undergone remodeling, leading to reprogramming. However, how cancer cells are reprogrammed and what the appropriate cues are remain largely unknown. Aberrant FA metabolism in cancer has also been correlated with malignant phenotypes, poor prognosis and chemoresistance. Dysregulation of FA metabolism not only accumulates FAs, but also generates extra metabolic intermediates, which may be utilized as signaling molecules for enhancing oncogenic signaling. In the previous sections, we summarized irregular FA metabolism in CSCs/TICs. However, the exact mechanism of FA metabolism in regulating CSCs/TICs/DTPs survival and expansion remains unclear. Understanding whether FAs serve as building blocks for CSCs/TICs/DTPs and/or whether the metabolic intermediates generated from FA metabolism are important signaling molecules for maintaining CSCs/TICs/DTPs or reprogramming cancer cells to CSCs/TICs/DTPs has implications for combating cancer therapeutic resistance.

How FA metabolism is regulated in CSCs also remains an outstanding question. Since FAs are not only important nutrients in human metabolism but also play a significant role in the composition of lipid bilayer membranes, it is likely that FA metabolism determines cell fate in a growing number of physiological and pathological conditions. The therapeutic manipulation of FAO holds great promise for the diagnosis and treatment of a wide range of human diseases in clinical settings. The master regulator of FA synthesis, SREBP1, regulates FASN expression to activate FA synthesis in cancer cells [1]. However, very little is known about the role of SREBP1 in CSCs/TICs/DTPs. SREBP1 binds to c-Myc to promote pluripotent gene expression in somatic cells [103], suggesting a potential role for SREBP1 in promoting cancer stemness. In breast cancer cells, leptin and transforming growth factor β (TGFβ) co-regulate AMPK-mediated ACC phosphorylation, implying that FAO is also affected by these signals [58]. Leptin signaling can also increase *CPT1B* expression via the JAK/STAT3 pathway to promote FAO [78]. Both leptin and TGFβ are secreted by adipose tissue [104–106], suggesting that FA metabolism in cancer cells may be regulated by the surrounding adipose tissue. Indeed, obesity has been associated with increased cancer risk and tumor progression [107, 108]. It is possible that adipose tissue in the tumor microenvironment secretes hormones and growth factors to reprogram FA metabolism in cancer cells and to drive cancer cell plasticity and promote cancer stemness.

Inhibition of FASN reduces numbers of CSCs [83, 85, 86], suggesting that FA synthesis is important for CSC maintenance, but how FAs facilitate CSC survival and expansion is unknown. Unsaturated FAs accumulate in ovarian CSCs and can activate NF-κB to regulate downstream stemness gene expression [89]. However, the detailed mechanism of how these unsaturated FAs activate NF-κB remains unclear. The specific roles of various types of FAs in maintaining CSCs/TICs/DTPs are also unknown. Future studies could use lipidome analysis to identify the composition of FA species and their function in CSCs/TICs/DTPs. Further efforts focusing on the identification and quantification of many metabolites from FA metabolism in a biological sample as possible will serve as a translatable tool to provide personalized medicine for individuals.

We suggest that preclinical and clinical studies are needed to address several key mitochondrial FAO-related questions. The first question is why and how does FAO enable the survival of CSCs/TICs/DTPs. We posit that FAO could serve three purposes: first, as a means to reduce lipotoxicity from lipid intermediates [109]; second, to energetically and efficiently generate ATP (e.g. in long-lived cell types, such as memory T cells, that depend on FAO for survival [110]; and third, to contribute to the accumulation of acetyl-CoA in the cytoplasm for protein acetylation and FA synthesis. It is still not fully understood why CSCs/TICs/DTPs rely on FAO for survival. A possible explanation is that during the process of FAO, an increase of NADPH and ATP helps CSCs/TICs/DTPs to survive. Elevated ROS is detrimental to CSCs/TICs/DTPs [111, 112] and NADPH serves as an antioxidant to reduce ROS levels. Consistent with this, inhibition of FAO reduces NADPH and ATP, leading to an increase of ROS and cell death in glioma [113]. Another possibility is that increased FAO generates increased oxidized nicotinamide adenine dinucleotide (NAD^+^), a cofactor for sirtuins (SIRTs). SIRT1–7 activity is regulated by the NAD^+^/NADH ratio. This family of deacetylases plays an important role in regulating stemness, tumorigenesis and many other critical cellular processes [114]. Blocking FAO by ETO results in decreased NAD^+^/NADH ratio and SIRT1 activity [115].

The next question is how does FA metabolism participate in the reprogramming process from cancer cells to CSCs/TICs/DTPs. Acetyl-CoA is a central metabolic intermediate at which multiple metabolic pathways converge. It is critical for initiating de novo FA synthesis and for incorporation into the TCA cycle to generate energy following FAO. Acetyl-CoA can also be an important source of histone or protein acetylation, which regulates a wide range of gene expression and protein functions. Acetyl-CoA homeostasis is controlled by several key enzymes. ACLY responsible for converting glucose-derived citrate into acetyl-CoA, which then affects histone acetylation to regulate gene expression [116]. ACC1 phosphorylation, which results in ACC1 inhibition, leads to accumulation of cytosolic acetyl-CoA. Accumulated acetyl-CoA causes total protein acetylation, including acetylation of the signal transducer Smad2; this enhances Smad2 transcriptional activity and ultimately results in EMT and metastasis in breast cancer [58]. FAO-derived acetyl-CoA can acetylate mitochondrial proteins, but the function of this phenomenon remains unknown [117]. Moreover, acetylation of ACSS2 inhibits its activity; SIRT3 can reverse the acetylation and activate ACSS2 [118]. Cancer cells preferentially utilize acetate as their carbon source, not only for FA synthesis, but also for epigenetic regulation via modulation of histone acetylation and associated gene expression. ACSS2 plays an important role in converting acetate into acetyl-CoA. Therefore, it is also involved in acetate-mediated epigenetic regulation [119]. For example, ACSS2 is phosphorylated at serine 659 by AMPK under metabolic stress and translocated to nucleus to locally produce acetyl-CoA for histone acetylation at the promoter regions of genes involved in autophagosome and lysosome formation [120]. That study provides strong evidence linking metabolism to epigenetic regulation of gene expression.

Another intriguing question is how are differentiated cancer cells reprogrammed into a stem-like or drug-tolerant state and what signals drive the process of reprogramming. Acetyl-CoA-mediated histone acetylation is controlled by glucose availability in embryonic stem (ES) cells and is responsible for maintaining the pluripotency of ES cells [121]. However, the gene expression profile associated with histone acetylation has not been revealed. Not only glucose, but also lipids, can be metabolized into acetyl-CoA, which then becomes a major carbon source for histone acetylation [122]. Moreover, the enhancement of both FAO and FA accumulation in breast cancer cells is linked to the acquisition of stem-like properties [94], implying that maximally functioning FA catabolism and anabolism may continuously provide acetyl-CoA for chromatin remodeling and reprogramming. Taken together, this suggests that lipid-derived acetyl-CoA is a major signaling metabolite that can reprogram cancer cells to acquire malignant phenotypes. Blockade of FAO with CPT inhibitors (e.g. ETO or perhexiline) or combination of FAO inhibitors with FASN inhibitors may hold hope for combating therapeutic resistance by eliminating CSCs/TICs/DTPs.

**Fig. 2:**
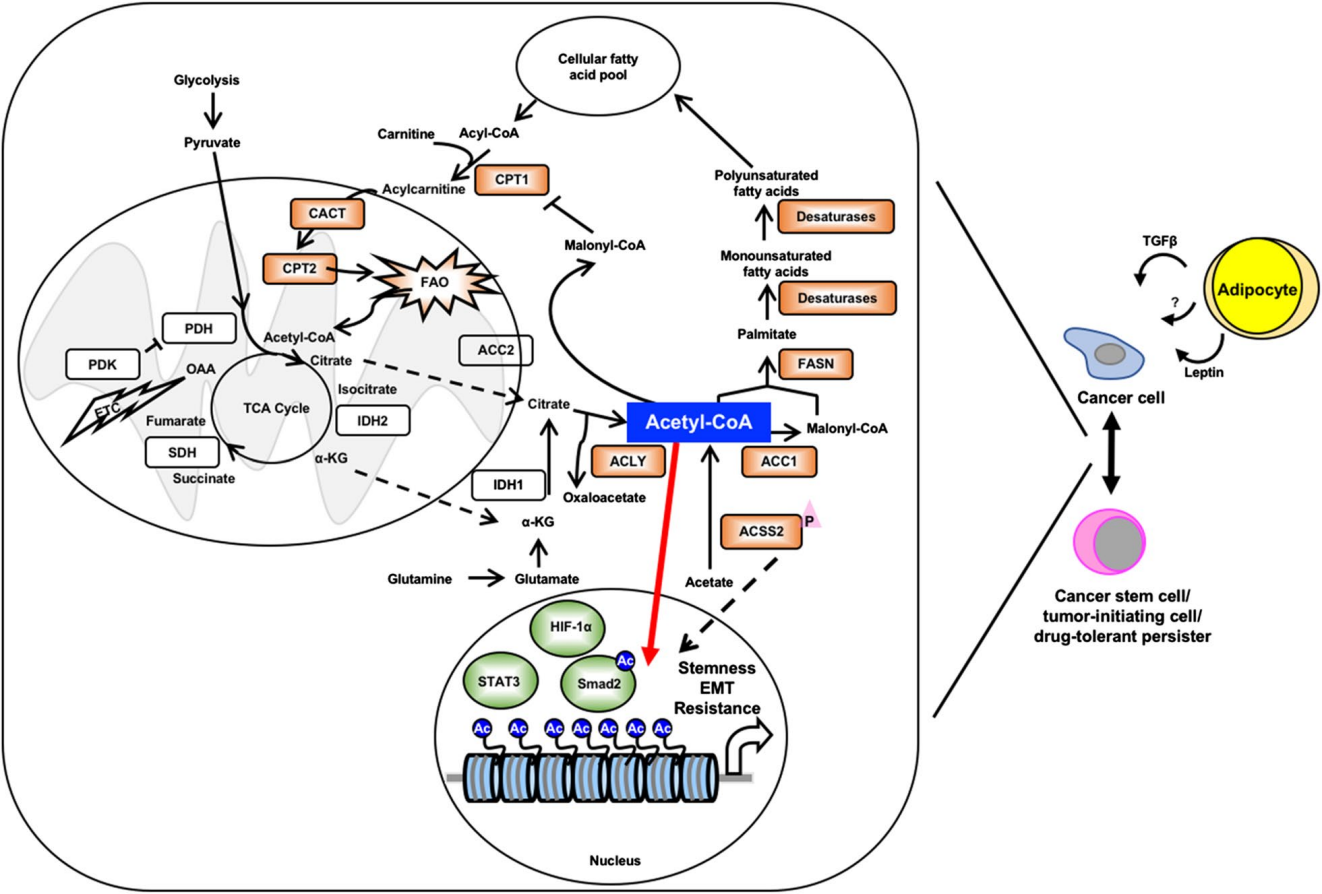
Potential roles of fatty acid metabolism in regulating cancer cell plasticity. Cancer cells can be reprogrammed into a cancer stemness state or drug-tolerant state with appropriate cues. It has been shown that adipocytes in the tumor microenvironment secrete leptin, transforming growth factor β (TGFβ) or other hormones and growth factors that support conversion of cancer cells into more malignant cell types, including cancer stem cells/tumor-initiating cells or drug-tolerant persisters. Acetyl-CoA is a central hub for multiple metabolic pathways including FA synthesis and FAO. Therefore, acetyl-CoA might be a major carbon source for histone acetylation and regulating gene expression for reprogramming. ACSS2 is phosphorylated and transferred to nucleus for histone acetylation. Some transcription factors, including hypoxia inducible factor-1α (HIF-1α), signal transducer and activator of transcription 3 (STAT3) and SMAD family member 2 (Smad2), are also involved in the conversion and may drive cancer cell plasticity

Lastly, both the lipogenic phenotype and cancer stemness can be induced by hypoxia [94, 123–126], suggesting that hypoxic signaling could be a converging pathway for both phenotypes. HIF-1α is the major regulator of hypoxic signaling, and a hypoxia- or pseudohypoxia-induced lipogenic phenotype can be HIF-1α-dependent [94, 126]. Moreover, HIF-1α induces expression of stemness factors, including Oct-4 and NANOG, and cancer cell plasticity observed in breast cancer is also dependent on HIF-1α [94, 125]. Therefore, HIF-1α may be an ideal target for shutting down both FA metabolism and stemness signaling in cancer cells, and ultimately preventing the conversion from cancer cells to CSCs/TICs/DTPs (Fig. 2).

## Conclusions

FA metabolism has drawn increasing attention in recent years. Particularly, the association between FA synthesis and the resulting lipogenic phenotype with cancer progression has been well-documented. However, fewer studies have focused on the role of FAO in CSCs/TICs/DTPs. Here, we have summarized evidences showing the relationship among FA metabolism, cancer stemness and therapeutic resistance and also discussed potential issues that may warrant further investigations. In the future, with more detailed mechanistic findings, therapeutic targeting of FA metabolism may be used to eradicate CSCs/TICs/DTPs and combat cancer more effectively.

## Abbreviations

ACC: acetyl-CoA carboxylase
ACLY: ATP-citrate lyase
ACSS2: acyl-CoA synthetase short-chain family member 2
AMPK: AMP-activated kinase
CACT: carnitine acylcarnitine translocase
CPT1: carnitine/palmitoyl-transferase 1
CSC: cancer stem cells
DKG: dimethyl α-ketoglutarate
DTP: drug-tolerant persisters
EMT: epithelial–mesenchymal transition
ES: embryonic stem
ETO: etomoxir
FA: fatty acid
FADH2: flavin adenine dinucleotide
FAO: fatty acid oxidation
FASN: fatty acid synthase
HER2: human epidermal growth factor receptor 2
HIF1α: hypoxia-inducible factor 1α
JAK: janus kinase
NAD^+^: oxidized nicotinamide adenine dinucleotide
NADH: reduced nicotinamide adenine dinucleotide
NADPH: nicotinamide adenine dinucleotide phosphate
OXPHOS: oxidative phosphorylation
PPAR: peroxisome proliferator-activated receptors
ROS: reactive oxygen species
SIRT: sirtuin
SREBP1: sterol regulatory element-binding protein 1c
STAT3: signal transducer and activator of transcription 3
TCA cycle: tricarboxylic acid cycle
TGF: transforming growth factor
TIC: tumor-initiating cells
TNBC: triple-negative breast cancer
